# Systemic therapies in advanced epithelioid haemangioendothelioma: A retrospective international case series from the World Sarcoma Network and a review of literature

**DOI:** 10.1002/cam4.3807

**Published:** 2021-03-13

**Authors:** Anna M. Frezza, Vinod Ravi, Salvatore Lo Vullo, Bruno Vincenzi, Francesco Tolomeo, Tom Wei‐Wu Chen, Pawel Teterycz, Giacomo G. Baldi, Antoine Italiano, Nicolas Penel, Antonella Brunello, Florance Duffaud, Nadia Hindi, Shintaro Iwata, Alannah Smrke, Alexander Fedenko, Hans Gelderblom, Winette Van Der Graaf, Aurore Vozy, Elizabeth Connolly, Massimiliano Grassi, Robert S. Benjamin, Javier‐Martin Broto, Giovanni Grignani, Robin L. Jones, Akira Kawai, Andrzej Tysarowski, Luigi Mariani, Paolo G. Casali, Silvia Stacchiotti

**Affiliations:** ^1^ Department of Medical Oncology IRCCS Fondazione Istituto Nazionale Tumori Milano Italy; ^2^ Department of Sarcoma Medical Oncology The University of Texas MD Anderson Cancer Center Houston Italy; ^3^ Unit of Clinical Epidemiology and Trial Organization IRCCS Fondazione Istituto Nazionale Tumori Milano Italy; ^4^ Department of Medical Oncology Università Campus Bio‐Medico di Roma Roma Italy; ^5^ Division of Medical Oncology Candiolo Cancer Institute FPO ‐ IRCCS Candiolo Torino Italy; ^6^ Department of Oncology National Taiwan University Hospital and Graduate Institute of Oncology National Taiwan University College of Medicine Taipei Taiwan; ^7^ Department of Soft Tissue/Bone Sarcoma and Melanoma Maria Sklodowska‐Curie National Research Institute of Oncology Warsaw Poland; ^8^ Department of Medical Oncology Nuovo Ospedale "S.Stefano" Prato Italy; ^9^ Early Phase Trials and Sarcoma Units Institut Bergonié Bordeaux France; ^10^ Medical Oncology Department Centre Oscar Lambret Lille France; ^11^ Medical School Lille University Lille France; ^12^ Department of Oncology Medical Oncology 1 Unit Istituto Oncologico Veneto IRCCS Padova Italy; ^13^ Department of Medical Oncology Medical Oncology La Timone University Hospital Aix‐Marseille Université (AMU Marseille France; ^14^ Medical Oncology Department University Hospital Virgen del Rocio, and Institute of Biomedicine Sevilla Spain; ^15^ Department of Musculoskeletal Oncology National Cancer Center Hospital Tokyo Japan; ^16^ Sarcoma Unit Royal Marsden NHS Foundation Trust/ Institute of Cancer Research, Chelsea London United Kingdom; ^17^ Division of Medical Oncology P.A. Herzen Cancer Research Institute Moscow Russian Federation; ^18^ Department of Medical Oncology Leiden University Medical Center Leiden the Netherlands; ^19^ Department of Medical Oncology Radboud University Medical Centre Nijmegen Nijmegen The Netherlands; ^20^ Department of Medical Oncology Pitié Salpétrière Hospital Paris France; ^21^ Department Of Medical Oncology Chris O'Brien Lifehouse Sydney Australia; ^22^ Medical Oncology Unit 1 Ospedale Policlinico San Martino IRCCS University of Genoa Genoa Italy; ^23^ Pathology department Maria Sklodowska‐Curie National Research Institute of Oncology Warsaw Poland; ^24^ Department of Oncology and Hemato‐oncology University of Milan Milan Italy

**Keywords:** anthracycline, chemotherapy, epithelioid haemangioendothelioma, interferon, paclitaxel, pazopanib

## Abstract

**Background:**

This observational, retrospective effort across Europe, US, Australia, and Asia aimed to assess the activity of systemic therapies in EHE, an ultra‐rare sarcoma, marked by *WWTR1*‐*CAMTA1* or *YAP1*‐*TFE3* fusions.

**Methods:**

Twenty sarcoma reference centres contributed data. Patients with advanced EHE diagnosed from 2000 onwards and treated with systemic therapies, were selected. Local pathologic review and molecular confirmation were required. Radiological response was retrospectively assessed by local investigators according to RECIST. Progression free survival (PFS) and overall survival (OS) were estimated by Kaplan‐Meier method.

**Results:**

Overall, 73 patients were included; 21 had more than one treatment. Thirty‐three patients received anthracyclines regimens, achieving 1 (3%) partial response (PR), 25 (76%) stable disease (SD), 7 (21%) progressive disease (PD). The median (m‐) PFS and m‐OS were 5.5 and 14.3 months respectively. Eleven patients received paclitaxel, achieving 1 (9%) PR, 6 (55%) SD, 4 (36%) PD. The m‐PFS and m‐OS were 2.9 and 18.6 months, respectively. Twelve patients received pazopanib, achieving 3 (25%) SD, 9 (75%) PD. The m‐PFS and m‐OS were.2.9 and 8.5 months, respectively. Fifteen patients received INF‐α 2b, achieving 1 (7%) PR, 11 (73%) SD, 3 (20%) PD. The m‐PFS and m‐OS were 8.9 months and 64.3, respectively. Among 27 patients treated with other regimens, 1 PR (ifosfamide) and 9 SD (5 gemcitabine +docetaxel, 2 oral cyclophosphamide, 2 others) were reported.

**Conclusion:**

Systemic therapies available for advanced sarcomas have limited activity in EHE. The identification of new active compounds, especially for rapidly progressive cases, is acutely needed.

## Graphical abstract

Anthracycline‐based regimens, the standard first line treatment in advanced soft tissue sarcomas, paclitaxel and pazopanib have a very limited activity in advanced, progressive epithelioid haemangioendothelioma. In the absence of available, potentially active medical therapies, epithelioid haemangioendothelioma remains today an unmet clinical need.

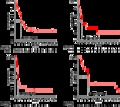

## 1 INTRODUCTION

Compared to other vascular sarcomas, epithelioid haemangioendothelioma (EHE) is marked by peculiar clinical features, defined molecular characteristics and specific treatment challenges.^1^

EHE is rarer than angiosarcoma or other vascular sarcomas (incidence rate <1/1,000,000), is more common in females and is often multifocal and/or multicentric at presentation, with lung, liver and bone being the typically involved sites.^2^ Despite its rarity, it encompasses a wide heterogeneity in clinical behaviour, with cases naturally stable over time as opposed to others which are slowly progressive. A rapidly evolving third variant does exist, which behaves as a high‐grade sarcoma. Similarly, the symptoms burden in this disease can be variable, but overall, a significant impact on quality of life and psychological distress have been recently reported in EHE patients.^3^

Serosal effusion and bone metastases have been reported as adverse prognostic factors.^2^, ^4^, ^5^ From the molecular point of view, the *WWTR1* *(WW* *Domain* *Containing* *Transcription* *Regulator* *1)* ‐ *CAMTA1* *(Calmodulin* *Binding* *Transcription* *Activator* *1)* and *YAP* *(Yes*‐*associated* *protein* *1)*‐*TFE3* *(Transcription* *Factor* *Binding* *To* *IGHM* *Enhancer* *3)* rearrangements are found in approximately 90% and 10% of all cases, respectively.^6^, ^7^ Recently, gene fusions involving *WWTR1* with a partner different from *CAMTA1* have also been reported.^8^ The detection of these rearrangements is today a hallmark in diagnosis, all the more in those cases marked by aggressive morphological features, where the differential diagnosis with angiosarcoma can be challenging, or atypical presentations. Both *WWTR1* and *YAP* are downstream effectors in the Hippo pathway, which could be therefore involved in the pathogenesis of this rare condition.^9^

In patients with asymptomatic, naturally stable, advanced EHE, watchful waiting is today a reasonable approach, as prolonged stabilities over time or even spontaneous regressions have been reported.^10^, ^11^, ^12^ Conversely, for symptomatic patients or patients with progressive multifocal or multicentric disease, systemic treatments are the standard of care. Liver transplant in patients with multifocal disease confined to the liver has also been investigated, but results are controversial.^11^, ^12^ Data currently available on systemic agents are limited to case reports or small single‐institution case series. Anecdotal responses have been reported with pazopanib, which is currently approved in the treatment of advanced soft tissue sarcomas refractory to anthracycline, as well as with other anti‐angiogenic compounds including sorafenib, bevacizumab and apatinib.^13^, ^14^, ^15^, ^16^, ^17^, ^18^ mTOR inhibitors (i.e. sirolimus) can control the disease in slowly progressive cases, whereas their activity seems to be limited in more aggressive variants, such as those presenting with serosal effusions.^5^, ^19^, ^20^ In the absence of any alternative potentially active treatment approved other that pazopanib, EHE patients with advanced progressive disease are treated with conventional chemotherapy, which is assumed to have very limited activity.^13^, ^21^

In this international, observational retrospective effort, we collected all the cases of histologically proven, advanced EHE, diagnosed from 2000 onwards and treated with systemic therapies at the major sarcoma reference centres within the framework of the *World* *Sarcoma* *Network*, with the aim of studying their potential. The results are reported herein.

## 2 PATIENTS AND METHODS

### 2.1 Patient population

We considered all patients of any age with advanced EHE, diagnosed from 2000 onwards, and treated with systemic therapies (anthracycline‐based regimens, weekly paclitaxel, pazopanib, INF‐α 2b or other), in front or further line. Patients treated sirolimus were not included in this series, as most of them are the subject of a separate report.^5^ The positivity of either *WWTR1*‐*CAMTA1* or *YAP1*‐*TFE3* rearrangement, determined by FISH or by positive immunostaining for *CAMTA1* or *TFE3*, was required. Patients were treated at 20 sarcoma reference centres in EU, US, Australia, and Japan, within the *World* *Sarcoma* *Network*. Written informed consent to the treatment was obtained as required by local regulation. Approval of this retrospective case series analysis by the Institutional Review Board of each participant institution was required.

### 2.2 Study design and data collection

Data were extracted from clinical databases and confirmed through a review of patient files. A questionnaire was circulated among participating institutions, in order to explore the frequency of radiological assessment for patients on treatment, at participating centres. All institutions evaluated disease response radiologically every 2–4 months. All participating sites were asked to provide a retrospective local assessment of treatment response based on RECIST version 1.1.^22^ Data on radiologic evidence of any disease progression prior to commencing treatment were also collected for all cases with available scans.

Together with data collection, a literature search was conducted. We searched for articles written in English, published in PubMed from 1995 until September 2020 with the terms ‘epithelioid haemangioendothelioma’, ‘chemotherapy’, ‘anthracycline’, ‘paclitaxel’, ‘pazopanib’, ‘antiangiogenic’, ‘treatment’. The final reference list was defined on the basis of originality and relevance to the scope of this article.

### 2.3 Statistical analyses

Descriptive statistics and frequency tabulation were used to summarize patient and tumor characteristics. Overall response rate (ORR) was defined as the proportion of patients who achieved complete response (CR) or partial response (PR) by RECIST 1.1. The corresponding 95% confidence intervals (CIs) were calculated based on the binomial distribution.

Progression‐free survival (PFS) and overall survival (OS) were estimated using the Kaplan‐Meier method, and survival distributions by group were compared using the log‐rank test. PFS was calculated as the interval from the start of treatment to the date of the first documented evidence of progressive disease (PD) or death due to any cause or to the date of the last follow‐up. OS was calculated as the interval from the start of treatment to the time of death from any cause or to the date of the last follow‐up. A two‐sided *p* < 0.05 was considered statistically significant.

Statistical analyses were carried out with SAS (version 9.4, SAS Institute, Cary, NC, USA) and R software (version 3.6.1, R Foundation for Statistical Computing, Vienna, Austria).

## 3 RESULTS

### 3.1 Patient population

Seventy‐three patients with advanced EHE treated with systemic therapies were identified. The median follow‐up was 35.8 months (interquartile range, IQR, 17.5–93.3) and the median OS was 17.4 months (IQR, 9.3 – not evaluable, NE). Thirty‐three patients received anthracycline‐based regimens, 11 weekly paclitaxel, 12 pazopanib, 15 INF‐α 2b and 27 other agents. Twenty‐one patients received more than one of the selected treatments, and therefore are included in more than one group. The population characteristics are summarized in Table 1.

**TABLE 1:** Population characteristics

	Anthracycline based regimens	Paclitaxel	Pazopanib	INF	Others
Patients	33	11	12	15	27
Median follow up, months (IQR)^a^	33 (19.4‐89.5)	80 (27.2 – 93.3)	15.4 (11.3 ‐ 17.2)	35.8 (17.5 ‐ 98.4)	NA
Marker	69 (95%)				
CAMTA1‐WWTR1^b^	4 (5%)				
YAP1‐TFE3	69 (95%)				
Median age (IQR)	47 (34 – 61)	39 (33 ‐ 68)	46 (42 – 58)	46 (41 ‐ 50)	48 (36 – 60)
Gender					
M	15 (45%)	4 (36%)	4 (33%)	7 (47%)	12 (44%)
F	18 (55%)	7 (64%)	8 (67%)	8 (53%)	15 (56%)
Stage (treatment start)					
Locally advanced	6 (18%)	1 (9%)	1 (8%)	3 (20%)	4 (14%)
Metastatic	27 (82%)	10 (91%)	11 (92%)	12 (80%)	23 (86%)
Evidence of prior PD:					
Yes	19 (58%)	6 (55%)	10 (83%)	12 (80%)	24 (89%)
No	14 (42%)	5 (45%)	2 (17%)	3 (20%)	3 (11%)
Number of previous systemic therapies					
0	29 (88%)	8 (73%)	5 (42%)	13 (87%)	10 (37%)^c^
1	4 (12%)	3 (27%)	6 (50%)	1 (7%)	12 (44%)
≥ 2	—	—	1 (8%)	1 (7%)	5 (19%)

Abbreviation: NA, not applicable.

^a^ 73 unique patients.

^b^ IHC o molecular testing.

^c^ Referred to the 1st of the "other treatments" of each patient

### 3.2 Treatment response and outcome

#### 3.2.1 Anthracycline‐based group

Thirty‐three patients were included, all evaluable for response. The median follow‐up was 33 months (IQR, 19.4–89.5). In 19 patients (58%) there was evidence of PD prior to commencing treatment, in 14 (42%) there was not. Fifteen patients (45%) received anthracycline as single agent, 9 (27%) in combination with ifosfamide and 9 (27%) in combination with different compounds. Twenty‐nine patients (88%) received anthracycline as a first‐line, 4 (12%) as a second line treatment. At the time of the present analysis, 32 patients completed their treatment. Treatment was discontinued for PD in 15 (47%) cases, for toxicity in 2 (6%), for maximum cumulative anthracycline dose in 14 (44%), and for other reasons in 1 (3%).

The best RECIST response with anthracycline‐based regimens was 1 (3%) PR, 25 (76%) SD, and 7 (21%) PD. The ORR was 3% (95%CI: 0%‐16%). The patient who achieved a response was a 30‐year old female, with a CAMTA1 positive EHE involving liver, lung, spleen and lymph nodes, treated with liposomal doxorubicin as a first‐line treatment. Details on the evidence or radiological disease progression prior to commencing treatment are reported in Tables 1 and 2.

**TABLE 2:** Treatment details and response

	Number of previous systemic therapies	Regimen	Disease response (RECIST 1.1)
Anthracycline‐based regimens (N = 33)	•0: 29 (88%) •1: 4 (12%) •≥ 2: 0	Anthracycline single agent: 15 (45%) Anthracycline+ifosfamide: 9 (27%) Anthracycline+others: 9 (27%)	CR: 0 PR: 1 (3%, *prior* *PD*) SD: 25 (76%) *Prior* *PD*: 14 *No* *prior* *PD*: 11 PD: 7 (21%)
Paclitaxel (N = 11)	•0: 8 (73%) •1: 3 (27%) •≥ 2: 0	Weekly paclitaxel: 11 (100%)	CR: 0 PR: 1 (9%, *no* *prior* *PD*) SD: 6 (55%) *Prior* *PD*: 2 *No* *prior* *PD*: 4 PD: 4 (34%)
Pazopanib (N = 12)	•0: 5 (42%) •1: 6 (50%) •≥ 2: 1 (8%)	NA	CR: 0 PR: 0 SD: 3 (25%) *Prior* *PD*: 2 *No* *prior* *PD*: 1 PD: 9 (75%)
INF‐α 2b (N = 15)	•0: 13 (87%) •1: 1 (7%) •≥ 2: 1 (7%)	NA	CR: 0 PR: 0 SD: 3 (25%) *Prior* *PD*: 2 *No* *prior* *PD*: 1 PD: 9 (75%)

Abbreviations: CR, complete response; NA, not applicable; NE, not evaluable; PD, progressive disease; PR, partial response; SD, stable disease.

**FIGURE 1:**
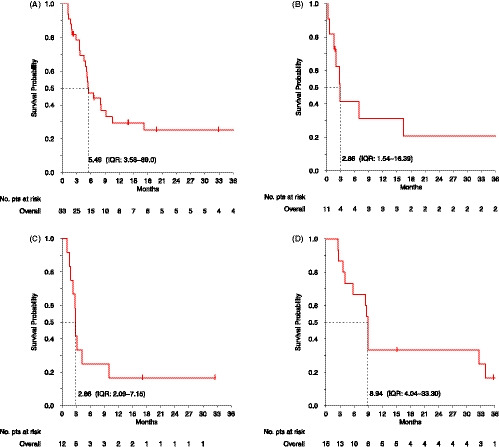
Kaplan‐Meier curves for progression‐free survival on anthracycline‐based regimens (A), paclitaxel (B), pazopanib (C) and *INF*‐*α* *2b* (D)

**FIGURE 2:**
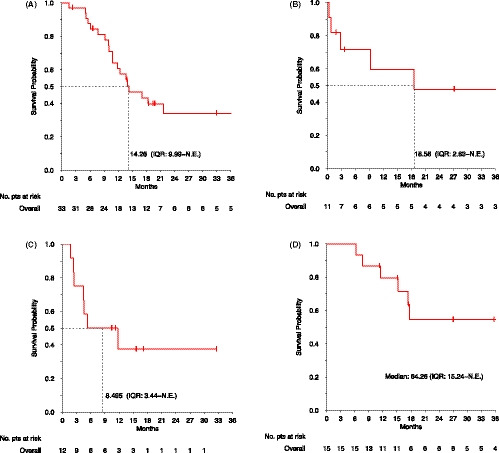
Kaplan‐Meier curves for overall survival on anthracycline‐based regimens (A), paclitaxel (B), pazopanib (C) and *INF*‐*α* *2b* (D)

The median PFS (m‐PFS) in this group was 5.5 months (IQR, 3.6 – 89.0, Figure 1A), and the median OS (m‐OS) 14.3 months (IQR, 10.0 ‐ NE, Figure 2A). The 3‐year PFS and OS rates were 25% (95% CI 13%‐48%) and 34% (95% CI 20%‐58%), respectively. No significant differences were noticed between patients with and without evidence of previous progression in terms of m‐PFS (5.4 and 5.5 months, respectively, *p* = 0.62), or m‐OS (13.7 and 18.4 months, respectively, *p* = 0.45).

#### 3.2.2 Weekly‐paclitaxel group

Eleven patients were included, all evaluable for response. The median follow‐up was 80 months (IQR, 27.2 – 93.3). In 5 patients (45%) there was evidence of PD prior to commencing treatment, in 6 (55%) there was not. Eight patients (73%) received weekly paclitaxel as a first‐line, 3 (27%) as a second‐line treatment. At the time of the present analysis, all patients completed their treatment. Treatment was discontinued for PD in 7 (64%) cases, for toxicity in 1 (9%), for agreement on a treatment holiday in 2 (18%), and for other reasons in 1 (9%).

The best RECIST response with weekly paclitaxel was 1 (9%) PR, 6 (55%) SD, and 4 (36%) PD. The ORR was 9% (95%CI: 0%‐41%). The patient who achieved a response was a 38‐year old male, with a YAP1‐TFE3 positive EHE of the lungs with pleural and lymph nodal involvement, treated with weekly paclitaxel as a first‐line treatment. Details on the evidence or radiological disease progression prior to commencing treatment are reported in Tables 1 and 2.

The m‐PFS in this group was 2.9 (IQR, 1.5 – 16.4, Figure 1B), and the m‐OS was 18.6 (IQR, 2.8 – NE, Figure 2B). The 3‐year PFS and OS rates were 21% (95% CI: 6%‐71%) and 48% (95% CI: 24%‐95%). In patients with no evidence of previous progression, compared to those who previously progressed, a trend toward a longer m‐PFS (2.0 and 6.9 months, respectively, *p* = 0.09) and a longer m‐OS (9.2 months vs. NE, *p* = 0.03) were recorded.

#### 3.2.3 Pazopanib group

Twelve patients were included, all evaluable for response. The median follow‐up was 15.4 months (IQR, 11.3 – 17.2). In 10 patients (83%) there was evidence of PD prior to commencing treatment, in 2 (17%) there was not. Five patients (42%) received pazopanib as a first‐line, 6 (50%) as a second‐line treatment and 1 (8%) in further line. At the time of the present analysis, 11/12 (91.7%) of patients completed their treatment. Treatment was discontinued for PD in 10 (91%) cases, for other reasons in 1 (9%).

The best RECIST response with pazopanib was 3 (25%) SD, and 9 (75%) PD. Details on the evidence or radiological disease progression prior to commencing treatment are reported in Tables 1 and 2.

The m‐PFS in this group was 2.9 (IQR, 2.1 – 7.1, Figure 1C), and the m‐OS was 8.5 (IQR, 3.4 ‐ NE, Figure 2C). The 2‐year PFS and OS rates were 17% (95% CI: 5–59%) and 38% (95% CI: 17–84%). No significant differences were noticed between patients with and without evidence of previous progression in terms of m‐PFS (2.9 months and NE, respectively, *p* = 0.47) whereas a trend toward a better m‐OS (8.5 months and NE, respectively, *p* = 0.86) was reported.

#### 3.2.4 INF‐α 2b group

Fifteen patients were included, all evaluable for response. The median follow‐up was 35.8 months (IQR, 17.5 – 98.4). In 12 patients (80%) there was evidence of PD prior to commencing treatment, in 3 (20%) there was not. Thirteen patients (87%) received INF‐α 2b as a first‐line, 1 (7%) as a second line treatment and 1 (7%) in further line. At the time of the present analysis, all patients completed their treatment. Treatment was discontinued for PD in 11 (73%) cases, for clinical choice after prolonged stability in 2 (13%) and for drug unavailability in 2 (13%).

The best RECIST response with INF‐α 2b was 1 (7%) PR, 11 (73%) SD, and 3 (20%) PD. The ORR was 7% (95%CI: 0%‐32%). The patient who achieved a PR was a 48‐year old male, with a WWTR1‐CAMTA positive EHE involving liver and lungs treated with INF‐α 2b as a first‐line treatment. Details on the evidence or radiological disease progression prior to commencing treatment are reported in Tables 1 and 2.

The median m‐PFS in this group was 8.9 months (IQR, 4.0 – 33.3, Figure 1D), and the m‐OS was 64.3 (IQR, 15.2 – NE, Figure 2D). The 3‐year OS and PFS rates were 17% (95% CI: 5–56%) and 54% (95% CI: 3–90%), respectively. In patients with no evidence of previous progression, compared to those who previously progressed, a trend toward a longer m‐PFS (32.6 vs. 8.8 months, respectively, *p* = 0.36) and a trend toward a longer m‐OS (NE vs. 64.3 months, *p* = 0.91) were noticed.

#### 3.2.5 Other systemic regimens

Among 27 patients treated with other regimens, 1 PR (prior PD, high‐dose ifosfamide) and 9 SD (all with prior PD, 5 gemcitabine plus docetaxel, 2 oral cyclophosphamide, 2 others) were reported.

Kaplan‐Meier curves for PFS and OS on anthracycline‐based regimes, paclitaxel, pazopanib and interferon are reported in Figures 1 and 2, respectively. Results on treatment and outcome for each regimen, and association with prior evidence of PD, are summarized in Table 2.

## 4 DISCUSSION

This academic, multi‐institutional, international, retrospective study collected the largest series ever of patients affected by advanced EHE treated with systemic therapies. Seventy‐three patients (33 treated with anthracycline‐based regimens, 11 with weekly paclitaxel, 12 with pazopanib, 15 with INF‐α 2b and 27 with other agents were included. Anthracycline‐based regimens, the standard first‐line therapy in advanced soft tissue sarcomas, showed a minor activity in advanced EHE (ORR of 3%, m‐PFS of 5.5 months). Similarly, a limited activity was seen with weekly‐paclitaxel (ORR of 9%, m‐PFS of 2.9 months) and pazopanib (no responses, m‐PFS of 2.9 months), while INF‐α 2b, which is currently not approved in the disease, resulted in an ORR of 7% and a m‐PFS of 8.9 months.

Our study is limited by its retrospective nature, both in the collection of clinical data and assessment of tumor response. The retrospective design limited a robust assessment of disease progression prior to commencing treatment, which would have been extremely valuable given the unpredictable natural history of EHE, and did not allow a reliable collection of data on the impact of systemic therapies in symptoms control and quality of life. Also, the number of patients included in each treatment group is limited. Despite these limitations, this is the largest series of EHE published so far and it was collected through a worldwide effort, with the contribution of 20 sarcoma reference centres. Because all of the contributing institutions are part of a network dedicated to the research and care of sarcomas, a reasonable level of consistency in terms of procedures can be assumed (including the frequency of radiological assessments during treatment). The confirmation of diagnosis by an expert sarcoma pathologist, together with the determination of CAMTA‐1 / TFE‐3 status is a major strength of this study.

Unfortunately, we could not observe any relevant anti‐tumor activity from any of the agents used. Notably, the m‐PFS for most systemic therapies in this series was lower than 6 months. This points to a patient population selected for an aggressive behaviour at the time of commencing systemic therapy. This is in line with the tendency of all expert centres to delay any treatment until the development of clear‐cut symptomatic or radiological progression. Indeed, in the absence of any approved treatment option, patients with advanced, progressive EHE are currently managed predominantly with conventional chemotherapy and pazopanib, similar to most advanced STS subtypes. The results of previous studies on systemic treatments in advanced EHE are summarized in Table 3.

**TABLE 3:** Summary of previous studies on systemic treatments in advanced epithelioid haemangioendothelioma

	Study type	Number of patients	CAMTA1 / TFE3 assessment	Regimen	Prior progression	Disease response	PFS (months)
Idilman R et al. Oncology. 1997	Case report	1	No	Doxorubicin	No	PR	NR (progression‐free at 12 months)
Pinet C et al. Eur Respir J. 1999	Case report	1	No	Carboplatin +Etoposide	Yes	CR	NR (progression‐free at 18 months)
Kayler L et al. Transplantation, 2002	Case report	1	No	INF‐α 2a	Yes	PR	NR (progression‐free at 4 months)
Mascarenhas RC et al. Oncology. 2004	Case report	1	No	Thalidomide	Yes	SD	NR (progression‐free at 36 months)
Kelly H et al. Lancet Oncol. 2005	Case report	1	No	Liposomal doxorubicin	No	SD	14
Al‐Shraim M et al. J Clin Pathol. 2005	Case report	1	No	INF‐α	Yes	PD	2
Marsh Rde W et al. Breast J. 2005	Case report	1	No	INF‐α	Yes	CR	NA
Bölke E et al. Eur J Med Res. 2006	Case report	1	No	Thalidomide	No	PD	NA
Celikel C et al. APMIS. 2007	Case report	1	No	Cisplatin +Doxorubicin + Cyclophosphamide	No	SD	NR (progression‐free at 12 months)
Calabro L et al. J Exp Clin Cancer Res. 2007	Case report	1	No	INF‐α 2a	Yes	SD	NA
Kassam A et al. J Pediatr Hematol Oncol. 2008	Case report	1	No	Vinblastine +Celecoxib + Thalidomide	Yes	PD	NA
Belmont L. J Thorac Oncol. 2008	Case report	1	No	Carboplatin +paclitaxel + bevacizumab	Yes	PR	NA
Radzikowska E et al. Pneumonol Alergol Pol. 2008	Case report	1	No	INF‐α 2a	Yes	SD	3
Saleiro S. Rev Port Pneumo. 2008	Case report	1	No	INF‐α 2a	Yes	PD	NA
Shilling G et al. Clin Oncol (Meeting Abstracts), 2009	Case report	1	No	Adriamycin and ifosfamide	Yes	PD	NA
				Lenalidomide	Yes	PR	NA
Lopes T, Rev Port Pneumol, 2009	Case report	1	No	Carboplatin +etoposide + bevacizumab	Yes	PD	NA
Lee YJ, Yonsei Med J, 2009	Case report	1	No	Adriamycin +dacarbazine + ifosfamide	Yes	SD	NA
Wedmid A et al. Nat Rev Urol. 2009	Case report	1	No	Liposomal doxorubicin followed by EBRT	Yes	PR	NR (progression‐free at 18 months)
Pintoffl J et al. Anticancer Drugs. 2009	Case report	1	No	Doxorubicine +Ifosfamide	Yes	PD	3
				Gemcitabine	Yes	SD	72
Raphael C et al. J Med Case Rep. 2010	Case report	1	No	Thalidomide	Yes	SD	NR (progression‐free at 84 months)
Sumrall A et al. J Neuro Oncology. 2010	Case report	1	No	Doxorubicin	Yes	PR	NA
				Thalidomide	Yes	SD	NA
				INF α 2b	Yes	SD	NA
				Lenalidomide	Yes	SD	NA
Kim YH, J Thoracic Oncology, 2010	Case report	1	No	Carboplatin +paclitaxel + bevacizumab	Yes	PD	3
Mizota A. J Thorac Oncol. 2011	Case report	1	No	Carboplatin +paclitaxel + bevacizumab	Yes	PR	NA
Trautmann K et al. Acta Oncol. 2011	Case report	1	No	Bevacizumab	Yes	SD	NR (progression‐free at 16 months)
Salech F et al. Ann Hepatol. 2011	Case report	1	No	Thalidomide	No	SD	NR (progression‐free at 109 months)
Grenader T et al. J Clin Oncol. 2011	Case report	1	No	Liposomal doxorubicin	Yes	PR	67 (60 months off treatment)
Mir O et al. Eur J Cancer. 2011	Multicenter, retrospective	1	No	Oral cyclophosphamide +prednisolone	No	CR	NA
Lazarus A et al. Clin Respir J. 2011	Case report	1	No	Taxol +Bevacizumab	No	PD	NA
		1	No	Carboplatin +Etoposide + Bevacizumab	Yes	PD	NA
Cioffi A et al. Journal of Clinical Oncology suppl. 2011	Multicenter, retrospective	16	No	Anthracycline (+/‐ Ifosfamide)	No	ORR =0	4.8 (median PFS)
		6	No	Other cytotoxic	No	ORR =0	
		6	No	Sorafenib	No	ORR =0	
		2	No	Metronomic cyclophosphamide	No	BR =SD	
		2	No	Thalidomide	No	BR =SD	
		2	No	Imatinib	No	BR =SD	
Tolkach Y. Onkologie. 2012	Case report	1	No	Sunitinib	Yes	SD	NR (progression‐free at 36 months)
Bansal A et al. Lung. 2012	Case report	1	No	Doxorubicin	Yes	PD	4
Gaur E et al. Cancer Biol Med. 2012	Case report	1	No	Nab‐paclitaxel +bevacizumab	Yes	SD	NA
Sangro B et al, Rare Tumours, 2012	Case report	1	No	Sorafenib	Yes	SD	NA
Chevreau C et al. Cancer. 2013	Prospective, phase 2	15	No	Sorafenib	Yes	ORR =13.3% (2/15)	6 (median PFS)
Agulnik M et al. Annals of Oncology. 2013	Prospective, phase 2	7	No	Bevacizumab	No	ORR =29% (2/7)	9 (median PFS)
Lakkis z et al. J Hepatol. 2013	Case report	1	No	Metronomic cycplophosphamide	Yes	PR	5
				Paclitaxel	Yes	PD	NA
				Etoposide +ifosfamide + cisplatin	Yes	PD	NA
				Sunitinib	Yes	PD	NA
		1	No	Metronomic cycplophosphamide	Yes	PR	17
Demir L et al. J Cancer Res Ther. 2013	Case report	1	No	Doxorubicin	Yes	SD	4
Ye B et al. Oncol Lett. 2013		1	No	Cisplatin +Paclitaxel + Endostar	Yes	SD	3
	Case report	1	No	Carboplatin +Paclitaxel + Bevacizumab	No	SD	8
		1	No	Carboplatin +Paclitaxel	No	SD	10
Pallotti MC et al. World J Gastroenterol. 2014	Case report	1	No	Lenalidomide	Yes	SD	39
Yousaf N et al. Anticancer Research. 2015	Retrospective, single institution	19	No	IFN, weekly paclitaxel, 5‐FU, caelyx, celecoxib, celecoxib +lenalidomide, doxorubicin, imatinib, carboplatin and paclitaxel, cyclophosphamide and vinblastine, axitinib, cyclophosphamide and etoposide, ifosfamide and doxorubicin, thalidomide, axitinib, pazopanib, semaxinib, sunitinib.	No	BR =PR (celecoxib, 1 patient); SD (other regimens)	NA
Soape MP et al. Case Rep Gastrointest Med. 2015	Case report	1	No	Thalidomide	Yes	SD	10
Semenisty V et el. BMC cancer. 2015	Case report	1	No	Pazopanib	No	SD (metabolic PR)	NR (progression‐free at 26 months)
Bally O et al. Clin Sarcoma Res. 2015	Case report	1	No	Doxorubicin	Yes	SD	10
				Brostacilline	Yes	SD	21
				Pazopanib	Yes	SD	NR (progression‐free at 100 months)
Kobayashi N et al. Case Rep Oncolo. 2016	Case report	1	No	Sorafenib	Yes	PR	NR (progression‐free at 60 months)
Kanemura S et al. Respirol Case Rep. 2016	Case report	1	No	Carboplatin +pemetrexed + bevacizumab	No	SD	NR (progression‐free at 6 months)
Mcculloch M et al. Perm J. 2016	Case report	1	No	Carboplatin +etoposide	Yes	SD	120
Stacchiotti S et al. Ann Surg Oncol. 2016	Multicenter, retrospective	17	Yes (16/17)	Sirolimus (plasma level of 15–20 ng/dL)	Yes	ORR=6% (1/16)	12 (median PFS)
Kollar A et al. Acta oncologica.2017	Retrospective analysis of prospective studies	10	No	Pazopanib	Yes	ORR=20% (2/10)	26 (median PFS)
Zheng Z et al. Medicine (Baltimore). 2017	Case report	1	No	Apatinib 500 mg daily	Yes	BR =SD	2
Afrit M et al. Cancer Biol Med. 2017	Case report	1	No	Doxorubicin	No	SD	5
Hettmer S et al. Pediatr Blood Cancer. 2017	Case report	1	Yes	Vincristine +cyclophosphamide + doxorubicin +paclitaxel	No	PR SD	NA (9 cycles) NR (progression‐free at 21 months)
				Lenalidomide			
Shiba S et al. BMC Cancer, 2018	Multicenter, retrospective	10	No	Carboplatin +Paclitaxel + Bevacizumab (CPB); Paclitaxel; Pazopanib; Bevacizumab; Streptozocina; Cisplatin +Epirubicin + Bevacizumab (CEB)	No	BR =PR (CPB); SD (other regimens)	NA
Giancipoli RG et al. Medicine (Baltimore). 2018	Case report	1	No	Cyclophosphamide	No	NA	12
				Pazopanib	No	SD (complete metabolic response)	NR (progression‐free at 112 months, 6 years off treatment)
Engel ER et al. J Pediatr Hematol Oncol. 2019	Multicenter, retrospective	6	Yes (1/6)	Sirolimus	Yes	ORR=50% (3/6)	22 (median PFS)
Zhou X et al. Clin Respir J. 2020	Case report	1	No	Docetaxel +Gemcitabine	No	PD	NA
Sparber‐Sauer M et al. Pediatr Blood Cancer. 2020.	Retrospective analysis of prospective studies	6	Yes	VAIA / VAC / CEVAIE, paclitaxel lenalidomide, INF, pazopanib	No	ORR =0	NA

Abbreviations: BR, best response; NA, Not Available; NR, Not Reached; ORR, overall response rate; PD, Progressive Disease; PFS, Progression Free Survival; PR, Partial Response; SD, stable disease.

Actually, anthracyclines, as single agents or in combination with alkylating agents such as ifosfamide, are the standard first‐line treatment in advanced STS, with an ORR reported in the range of 20% and a m‐PFS in the range of 6 months across different studies, which is regarded as clinically meaningful in this setting, taking into consideration the biological aggressiveness of most STSs and their tendency toward a rapid progression.^23^, ^24^, ^25^ In advanced EHE, data on the activity of anthracycline‐based regimens are limited. To the best of our knowledge, only 2 responses have been reported with doxorubicin in 1997 and 2010, in two metastatic EHE patients, however, the diagnosis was not confirmed by either molecular testing or IHC.^26^, ^27^ Cioffi A. et al. did not record any radiological responses in 16 advanced EHE patients treated with anthracyclines, with a m‐PFS of 4.8 months.^19^ Similarly, no PR were reported by Yousaf N. et al and in most case reports available.^13^ Consistent with these findings and on a larger scale, our series confirmed the very limited activity of anthracycline in advanced EHE, with only 1 PR observed (ORR: 3%) and a m‐PFS of 5.5 months. Four patients were treated with liposomal doxorubicin, all with prior evidence of PD, and a PR and 2 SD were observed. In the literature, 3 case reports highlighting a PR and a prolonged disease control in advanced, rapidly progressive EHE treated with liposomal doxorubicin, are available. All this would suggest a possible role for this agent in the management of progressive EHE.^28^, ^29^, ^30^

Paclitaxel has a recognized activity in vascular sarcomas, especially in angiosarcoma, in which an ORR in the range of 20–50% across different series has been reported.^31^, ^32^ In advanced EHE, no responses to paclitaxel have been described in the few case‐reports available, nor in the retrospective series by Yousaf N. et al, where 6 patients received paclitaxel across different lines with a median treatment duration of 3 months.^13^ Consistently, only 1 PR was observed (ORR=9%) over 11 patients treated with weekly paclitaxel in this series, predominantly (73%) as a front‐line, with a m‐PFS of 2.9 months.

Responses to pazopanib in EHE have been described in 4 different clinical reports on previously progressive patients, among which a retrospective study from EORTC exploring the activity of pazopanib in a series of vascular sarcomas.^14^, ^15^, ^33^, ^34^ In this last study, 1 CR and 1 PR were observed over 10 advanced EHE treated.^15^ Conversely, no response was seen in the single case described by Yousaf N et al.^13^ Similarly, no responses were seen in our study of 12 EHE patients who received pazopanib, mostly in first and second line, with a short m‐PFS (2.9 months). Notably, in these previously published studies molecular confirmation of diagnosis is lacking.

The activity of INF‐α in advanced EHE has already been reported, being used both in the paediatric and adult population.^35^, ^36^, ^37^ The agent was used assuming an anti‐angiogenic effect. Though in our series only 1 PR was observed in 15 patients, m‐PFS was longer than any other agent, e.g. 8.9 months. Taken together, available results seem to confirm the potential role of interferon in the management of this rare sarcoma subtype, though the drug is not approved for this indication.

Similarly, sirolimus is also potentially active in the treatment of advanced EHE, with a reported ORR of 10% and a m‐PFS of 13 months in previously progressive patients.^5^ There are some patients with an aggressive behavior, often marked by a serosal effusion, who respond less to therapy.^5^ In this series, we did not to include patients treated with sirolimus, as they are the subject of a separate paper.

In brief, it is clear from this series, and the literature, that we lack any standard medical therapy in EHE. In particular, active drugs in STS are essentially inactive in EHE. An old agent such as IFN has some activity but is not approved for this disease. The same is true of other agents such as mTOR inhibitors, for which retrospective and anecdotal evidence points to slightly less than one half of previously progressing patients free of progression at two years.^5^, ^20^ Undoubtedly, the natural history and uncertain prognosis of these patients make clinical studies very difficult, while a proportion of them does not require any treatment. Indeed, controlled studies are virtually impossible due to the rarity of disease, but a distinct number of patients present with an aggressive clinical behavior and are in need for front‐line active treatments.^5^

Clearly, any prospective study on any new therapy would need a clear background landscape in terms of definition of the disease and its natural history. To this aim, a global consensus development process is in place. At least, it may clarify which subgroups of patients are more in need for any medical therapy and which is their expected prognosis. Indeed, the biomolecular profile of the disease is now better understood and at least is able to single out true EHE from other vascular sarcomas, primarily angiosarcoma. Failing to do so in the past was a major obstacle even to developing new therapies. Further understanding of the molecular pathogenesis might obviously help find new therapeutic targets. The Sarcoma Alliance for Research through Collaboration (SARC) have conducted a phase 2 trial of trametinib in unresectable/ metastatic EHE. Other global efforts are ongoing, and it is worth highlighting that they involve patient groups.

## 5 ETHICS STATEMENT

Approval of this retrospective case series analysis by the Institutional Review Board of each participant institution was required.

## CONFLICT OF INTEREST

*AMF* has received institutional research funding from Advenchen, Amgen Dompè, Bayer, Blueprint, Daiichi Sankyo, Epizyme, Eli Lilly, Glaxo, Karyopharm, Novartis, Pfizer, Pharmamar, Springworks; travel coverage by Pharmamar. *BV* has received institutional research grants from Eli Lilly, Novartis, PharmaMar; honoraria for advisory board participation from Eisai, Eli Lilly, Novartis, PharmaMar and Abbot; testimony fee from Abbot; research funding for clinical studies (institutional) from PharmaMar, Eli Lilly and Novartis. *GB* has received onoraria for consultancy from Eli Lilly, Eisai and PharmaMar; travel grants from PharmaMar, Pfizer and Eli Lilly; advisory board from AboutEvents, EditaMed, Eli Lilly. *AI* reports research grants from AstraZeneca, Bayer, Ipsen, Roche, MSD, Merck, Novartis, PharmaMar; honoraria for advisory board participation and expert testimony from Bayer, Daiichi SankyoRoche, Epyzime, Springworks, PharmaMar, Eli Lilly and Company. *AB* has received honoraria for consultancy or advisory board from Eli Lilly, Roche and Eisai; travel grants from PharmaMar and Ipsen. *NH* reports research grants from PharmaMar, Eisai, Immix BioPharma and Novartis outside the submitted work; honoraria and travel grants from PharmaMar; and research funding for clinical studies (institutional) from PharmaMar, Eli Lilly and Company, Adaptimmune Therapeutics, AROG, Bayer, Eisai, Lixte, Karyopharm, Deciphera, GSK, Novartis, Blueprint, Nektar, Forma, Amgen and Daiichi Sankyo. *WVDG* has received honoraria for advisory board participation from Bayer and GSK, consultant: Springworks, research project Novartis. *JMB* reports research grants from PharmaMar, Eisai, Immix BioPharma and Novartis outside the submitted work; honoraria for advisory board participation and expert testimony from PharmaMar, Eli Lilly and Company, Bayer and Eisai; and research funding for clinical studies (institutional) from PharmaMar, Eli Lilly and Company, Adaptimmune Therapeutics, AROG, Bayer, Eisai, Lixte, Karyopharm, Deciphera, GSK, Novartis, Blueprint, Nektar, Forma, Amgen and Daiichi Sankyo. *GG* has received research grant from Pharmamar and Bayer; honoraria for advisory board from Lilly, Pharmamar, Novartis, Merck, Bayer, EISAI. *RLJ* is the recipient of grants/research support from MSD, GSK. RLJ is the recipient of consultation fees from Adaptimmune, Athenex, Blueprint, Clinigen, Eisai, Epizyme, Daichii, Deciphera, Immunedesign, Lilly, Merck, Pharmamar, Tracon, UptoDate. *PR* has received honoraria for lectures and Advisory Boards from MSD, BMS, Novartis, Pierre Fabre, Merck, Sanofi and Blueprint Medicines outside the scope of the study. *SS* has received honoraria from Glaxo, Pharmamar; fee for advisory role from Bavarian Nordic, Bayer, Epizyme, Eli Lilly, Daiichi Sankyo, Deciphera, Glaxo, Maxivax, Pharmamar; institutional research funding from Advenchen, Amgen Dompè, Bayer, Blueprint, Daiichi Sankyo, Epizyme, Eli Lilly, Glaxo, Karyopharm, Novartis, Pfizer, Pharmamar, Springworks. SLV, LM, FT, TWC, PT, NP, AS, AF, HG, AT, EC, MG, AK, SI, FD, AV, VR, RSB have nothing to disclose.

## AUTHOR CONTRIBUTIONS

*Study* *concept* *and* *design*: Anna Maria Frezza, Silvia Stacchiotti. *Acquisition*, *analysis* *or* *interpretation* *of* *data*: Anna Maria Frezza, Vinod Ravi, Salvatore Lo Vullo, Bruno Vincenzi, Francesco Tolomeo, Tom Wei‐Wu Chen, Pawel Teterycz, Giacomo Giulio Baldi, Antoine Italiano, Nicolas Penel, Antonella Brunello, Florance Duffaud, Nadia Hindi, Shintaro Iwata, Alannah Smrke, Alexander Fedenko, Massimiliano Grassi, Hans Gelderblom, Winette Van Der Graaf, Aurore Vozy, Elizabeth Connolly, Robert S. Benjamin, Javier‐Martin Broto, Giovanni Grignani, Robin L. Jones, Akira Kawai, Piotr Rutkowski, Luigi Mariani, Paolo G. Casali, Silvia Stacchiotti. *Drafting* *of* *the* *manuscript*: Anna Maria Frezza, Vinod Ravi, Salvatore Lo Vullo, Bruno Vincenzi, Francesco Tolomeo, Tom Wei‐Wu Chen, Pawel Teterycz, Giacomo Giulio Baldi, Antoine Italiano, Nicolas Penel, Antonella Brunello, Florance Duffaud, Massimiliano Grassi, Nadia Hindi, Shintaro Iwata, Alannah Smrke, Alexander Fedenko, Hans Gelderblom, Winette Van Der Graaf, Aurore Vozy, Elizabeth Connolly, Robert S. Benjamin, Javier‐Martin Broto, Giovanni Grignani, Robin L. Jones, Akira Kawai, Piotr Rutkowski, Luigi Mariani, Paolo G. Casali, Silvia Stacchiotti. *Critical* *revision* *of* *the* *manuscript* *for* *important* *intellectual* *content* **:** Anna Maria Frezza, Vinod Ravi, Salvatore Lo Vullo, Bruno Vincenzi, Francesco Tolomeo, Tom Wei‐Wu Chen, Pawel Teterycz, Giacomo Giulio Baldi, Antoine Italiano, Nicolas Penel, Massimiliano Grassi, Antonella Brunello, Florance Duffaud, Nadia Hindi, Shintaro Iwata, Alannah Smrke, Alexander Fedenko, Hans Gelderblom, Winette Van Der Graaf, Aurore Vozy, Elizabeth Connolly, Robert S. Benjamin, Javier‐Martin Broto, Giovanni Grignani, Robin L. Jones, Akira Kawai, Piotr Rutkowski, Luigi Mariani, Paolo G. Casali, Silvia Stacchiotti. *Statistical* *analysis*: Salvatore Lo Vullo, Luigi Mariani. *Study* *supervision*: Anna Maria Frezza, Salvatore Lo Vullo, Luigi Mariani, Paolo G. Casali, Silvia Stacchiotti.

## ACKNOWLEDGEMENT

This study has been supported by the SARCOMICS INT 77/18 project.

## Data Availability

Data sharing is not applicable to this article as no new data were created or analyzed in this study.
